# Repeated Failure in Reward Pursuit Alters Innate *Drosophila* Larval Behaviors

**DOI:** 10.1007/s12264-018-0248-0

**Published:** 2018-06-27

**Authors:** Yue Fei, Dikai Zhu, Yixuan Sun, Caixia Gong, Shenyang Huang, Zhefeng Gong

**Affiliations:** 1 School of Life Sciences, Zhejiang University, Hangzhou, 310058 China; 2 Department of Neurobiology, Key Laboratory of Medical Neurobiology of the Ministry of Health of China, Key Laboratory of Neurobiology, Zhejiang University School of Medicine, Hangzhou, 310058 China; 3 Trinity College of Arts and Sciences, Duke University, Durham, NC 27708 USA

**Keywords:** *Drosophila* larva, Repeated failure in reward pursuit, Octopamine

## Abstract

Animals always seek rewards and the related neural basis has been well studied. However, what happens when animals fail to get a reward is largely unknown, although this is commonly seen in behaviors such as predation. Here, we set up a behavioral model of repeated failure in reward pursuit (RFRP) in *Drosophila* larvae. In this model, the larvae were repeatedly prevented from reaching attractants such as yeast and butyl acetate, before finally abandoning further attempts. After giving up, they usually showed a decreased locomotor speed and impaired performance in light avoidance and sugar preference, which were named as phenotypes of RFRP states. In larvae that had developed RFRP phenotypes, the octopamine concentration was greatly elevated, while *tβh* mutants devoid of octopamine were less likely to develop RFRP phenotypes, and octopamine feeding efficiently restored such defects. By down-regulating tβh in different groups of neurons and imaging neuronal activity, neurons that regulated the development of RFRP states and the behavioral exhibition of RFRP phenotypes were mapped to a small subgroup of non-glutamatergic and glutamatergic octopaminergic neurons in the central larval brain. Our results establish a model for investigating the effect of depriving an expected reward in *Drosophila* and provide a simplified framework for the associated neural basis.

**Electronic supplementary material:**

The online version of this article (10.1007/s12264-018-0248-0) contains supplementary material, which is available to authorized users.

## Introduction

Animals prefer to be rewarded. In a widely-used conditioned place-preference test, rodents prefer to stay in a place that is associated with rewards such as food or drugs like cocaine and morphine [1, 2]. Brain regions, including the nucleus accumbens and ventral tegmental area, have been suggested to be associated with reward. Reward is often used as a reinforcer in training an animal to learn a task. In a learned reward-enforced task, an animal shows an expectation-specific response to the reward before it is delivered. For example, in a delayed go-no-go task in monkeys, activation of certain neurons in anterior parts of the caudate nucleus, putamen, and ventral striatum occur before the delivery of a reward [3]. Expectation of a reward also shapes visual information-processing in monkeys [4]. So far, the neural bases of reward and expectation of reward have received much investigation in rodents and primates. Comparatively less is known about the effect of failure to get the expected reward, despite the fact that this process is normal in an animal’s life, such as failure to catch prey or in courtship. The purpose of this study was to investigate the effects of deprivation of an expected reward and the related neural and molecular mechanisms.

Octopamine, an arthropod homolog of norepinephrine in *Drosophila*, is a biological amine that works as a neurotransmitter/neuromodulator that regulates various aspects of *Drosophila* behaviors, such as aggression [5–8], sleep [9, 10], and the stress response [11–15]. Octopamine is also known to be a reward-signaling molecule that allows the appetitive reinforcement in classical olfactory conditioning in *Drosophila* [16–19]. Thus, octopamine is able to regulate mood-like behavior in *Drosophila* [20, 21]. The homolog of octopamine, norepinephrine, is known to be involved in mood modulation in vertebrates [22], but whether octopamine participates in reward deprivation-related behavior in *Drosophila* is not clear.

In this work, we adopted a strategy in which a *Drosophila* larva was kept away from an appetitive food or odor when it was about to reach the attractant and before it finally abandoned further attempts. Our results showed that a larva gave up chasing an attractant after 3 or 4 trials. After giving up, a larva usually showed a decreased locomotor velocity as well as impaired performance in light avoidance and sugar preference, which were called phenotypes of RFRP states. We also found that octopamine was required for the development RFRP states and the exhibition of RFRP phenotypes. A small group of neurons in the larval brain hemispheres and subesophageal ganglion (SOG) appeared to be the crucial octopamine-releasing sites for the regulation of larval RFRP phenotypes.

## Materials and Methods

### Fly Strains

The following fly strains were used: *WTB, w*^*1118*^*, Tdc2-Gal4, tβh*^*1*^*, vGlut-Gal4, vGlut-Gal80, NP7088-Gal4* [16]*, 0665-Gal4* [20]*, UAS-tβh-RNAi, UAS-Chrimson* [23], and *UAS-CaMPARI* [24]. The *WTB* and *tβh*^*1*^ mutants were outcrossed with *w*^*1118*^ for 5 generations to replace the genetic background before being used for comparison, except that the red-eye phenotype was retained. Flies were raised at 25°C on standard medium under a 12:12 h light/dark cycle as previously described [25].

### Food

Standard food was provided as described previously [25] unless otherwise stated.

To investigate the effect of drugs on larval RFRP-related phenotypes, octopamine (Cat#: O101521, Aladin Inc., Shanghai, China) at different concentrations was dissolved in water and added to standard fly food during preparation.

### Larval RFRP Training

Induction of larval RFRP phenotypes: Single third-instar larvae were rinsed with distilled water and placed on an agar plate on paper with a 1 cm × 1 cm grid. The larva was allowed to move freely for ~1 min to adapt to the new environment. An attractant source, such as butyl acetate (BA; B116225, Aladin Inc., Shanghai, China) dissolved in paraffin oil (30139828, Sinopharm Inc., Shanghai, China) at various concentrations was placed on a 1 cm × 1 cm × 0.2 cm plastic container on the agar plate and its edge was 1.5 cm from the larva. The larva moved toward the attractant source and when it was ~ 0.25 cm from the source, the container with the source was quickly moved 1.5 cm further from the larva, at an angle of 45^o^ – 90^o^ relative to the larval heading direction. If the larva continued to pursue the attractant, the container-moving process was repeated. If the larva was > 3 cm from the container, it was considered to have failed in its pursuit of the attractant and given up further attempts. For each round of container movement, the container was cleaned and another 100 μL BA dissolved in paraffin oil was added to keep the attractant concentration stable. The number of trials in which a larva attempted to reach the attractant before it finally gave up was scored to reflect its vulnerability. For larval RFRP training with paraffin oil, the procedure was the same except that no BA was dissolved in the oil. Also see supplementary Video 1. For control of BA exposure, ~ 2 mL pure BA was added to a 15 cm × 15 cm × 7 cm plastic capped box and 5 min was allowed for the BA vapor to fill the box. After that, an agar plate with a larva and a container with 1 mL pure BA 1.5 cm above the larva was placed in the box for 5 min.

For the yeast protocol, 1 g yeast was dissolved in 100 μL water. One drop of yeast solution was placed on an agar plate. A larva placed 1.5 cm away was attracted to the yeast and if it reached ~ 0.25 cm from the yeast, it was pulled back to the starting position until it gave up pursuit.

### Measuring Locomotor Velocity

Individual larvae were tested immediately after RFRP training. A larvae-containing agar plate was placed on a piece of black paper with white concentric circles at intervals of 0.25 cm. Larval displacements within 30 s were measured before RFRP training to obtain an estimate of velocity. In control groups, 30-s larval displacements were measured 5 min after the first measurements. In RFRP-trained larvae, 30-s larval displacements were measured immediately after the larva gave up pursuing the attractant. To normalize the change in velocity after RFRP training, we used the logarithm of the ratio of the velocity after RFRP to that before RFRP, log(*V*_RFRP_/*V*_ctr_), for statistical analysis.

### Measuring Larval Light Avoidance

Individual larvae after RFRP training were immediately tested. Light from a fluorescent white light source was projected through a hole in foil onto an agar plate to form a spot 1 cm in diameter at an intensity of 500 lux. An individual 3rd instar larva was placed at the margin of the spot, which was orientated in front of the larva. Then, it moved toward and entered the light spot. If the larva retreated or turned its head > 90^o^, a light-avoiding event was considered to have occurred. This test was repeated ten times at intervals of 20–30 s. The number of light-avoiding events was used to measure larval light avoidance.

### Sugar Preference Assay

A 1.5% agar plate containing 0.5 mol/L sucrose was prepared and cut into halves. One half was transferred into another empty Petri dish of the same size. Agar (1.5% *w*/*w*) was poured into the other half of the Petri dish to make an agar plate in which one half had sugar and the other half did not. An individual larva was placed on the boundary between the sugar and non-sugar halves and 5 min later, its position was recorded. The ratio of larvae on the non-sugar half was calculated.

### Evaluating Attractive Distance of BA

A group of 10 larvae was placed at various distances from the container with or without BA. Larvae that entered the circle at 0.25 cm from the container within 2 min were considered to be effectively attracted by BA.

### Liquid Chromatography–Mass Spectrometry

The brains of five 3rd instar larvae were quickly dissected, placed in 1.5 mL centrifuge tubes and mixed with 100 μL 50% acetonitrile on ice. Then they were ground (25/s for 2 min) and centrifuged at 18,000 rpm at 4°C for 15 min to remove the insoluble residue. Finally, the supernatant was used for testing in a 6400 Series Triple Quadrupole B.07.01 (B7112.0).

Ten microliters of supernatant containing tracer were injected by an autosampler onto an HPLC using a Zorbax SB C18 column (Part# 866953-902, Agilent Technologies, Wilmington, DE). Separation was achieved through gradient conditions. Until 2 min, the mobile phase was 95% acetonitrile containing 0.1% formic acid and 5% methanol. From 2 min to the end, the mobile phase was 5% acetonitrile containing 0.1% formic acid and 95% methanol. The flow rate was 0.3 mL/min. After HPLC, an API 3000 LC/MS/MS mass spectrometer (Applied Biosystems, Foster City, CA) in positive electrospray mode was applied using Multiple Reaction Monitoring (MRM) methods. The MS/MS system used polarity-switching electrospray ionization. The optimal conditions were capillary voltage, 3 kV (+); nebulization pressure, 45 psi; drying gas temperature 325°C; drying gas flow, 5 L/min; sheath gas temperature, 350°C; and sheath gas flow, 11 L/min. MRM transitions, collision energy, and other conditions are shown in Table 1.

**Table 1:** Parameters of different compounds in LC-MS

Compound name	Precursor ion (m/z)	Production (m/z)	Fragmentation voltage (V)	Collision energy (V)
5-HT	177.1	160	60	5
OA	154	137	60	7
DA	154	136	60	2
TA	138.1	121	60	7

*5-HT* serotonin, *DA* dopamine, *OA* octopamine, *TA* tyramine

### Microscopy

To visualize Gal4 expression patterns, larval brains were dissected in PBS, fixed in PBS containing 4% formaldehyde for 60 min at room temperature, and washed with PBS wih 1% Triton X-100 (PBT). Tissues were mounted and images were acquired using a FV1000 confocal laser scanning microscope and subsequently processed with ImageJ (http://imagej.nih.gov/ij/) and Photoshop (Adobe Inc., San Jose, CA).

## CaMPARI Imaging

A high-power 405-nm LED (Warsun, R838-4 LED, 80 mW) was positioned 5 cm above the larva on the agar plate. Larvae were subjected to RFRP training or 5-min exposure to 1:200 BA dissolved in paraffin oil in a 1 cm × 1 cm × 0.2 cm plastic container placed 1.5 cm above the larva. Then, the larvae were irradiated with ultraviolet light for 50 s. The brains were then quickly dissected in PBS without fixation before being mounted; images were acquired under the FV1000 microscope. Green-to-red photoconversion was quantified by analyzing the maximum green and red fluorescence intensity in each cell body in projections of the acquired z-stacks. Red fluorescence intensity was divided by green fluorescence intensity to obtain log (F_red_/F_green_).

### Statistics

For larval locomotion, the *t*-test or one-way ANOVA was used. For all the rest except for sugar preference, the non-parametric *Kruskal-Wallis* test was used for comparisons between > 3 groups, and the non-parametric Mann-Whitney test was used for comparisons between two groups. For sugar preference, Fisher’s exact test was used.

## Results

### RFRP Phenotypes in *Drosophila* Larvae

**Fig. 1:**
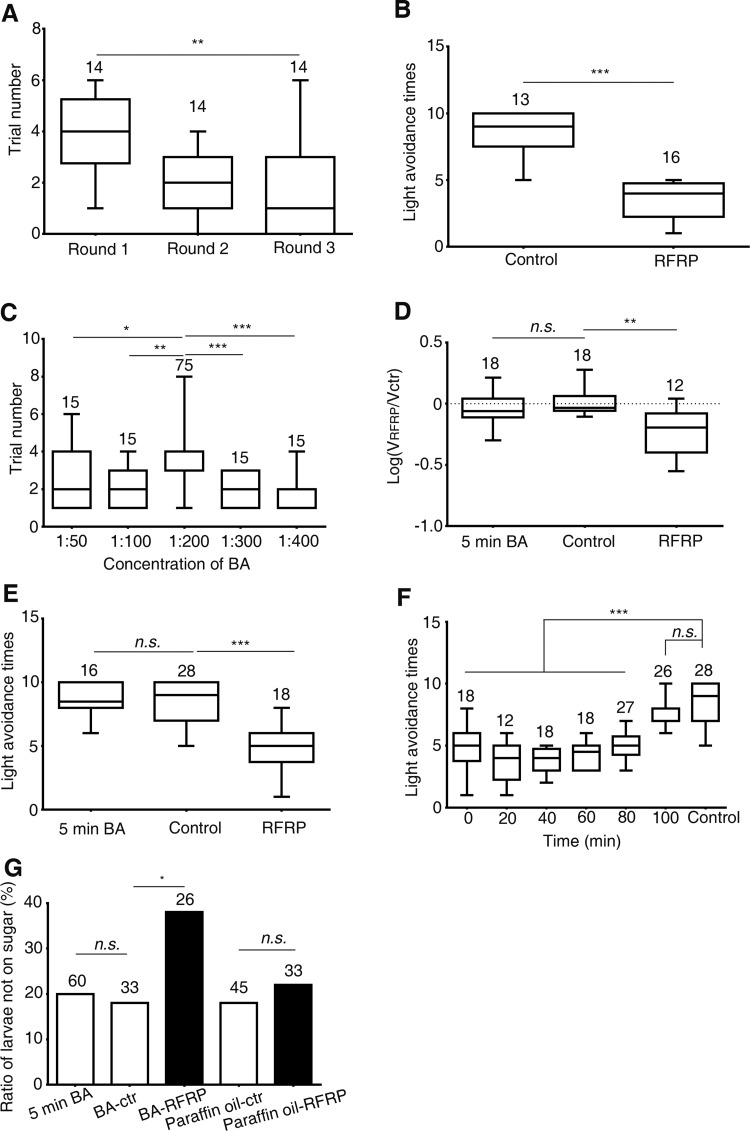
RFRP phenotypes are induced in *Drosophila* larvae by repeated failure to obtain a yeast or BA reward. **A** Number of trials to obtain a yeast reward decreased after RFRP training. Time interval between rounds was 10 min. **B** Larval light avoidance decreased after RFRP training. **C** Trial number in RFRP training with BA at different concentrations. A 1:200 dilution of BA yielded the highest trial number. **D** Locomotor velocity was significantly decreased after RFRP training with BA at 1:200. Exposing larvae to BA alone for 5 min did not affect locomotor velocity. **E** Larval light avoidance was decreased after RFRP training with BA at 1:200. Exposing larvae to BA for 5 min did not affect light avoidance. **F** Decrease in larval light avoidance after RFRP training with BA at 1:200 lasted for at least 60 min. **G** Larval preference for sugar was decreased after RFRP training with BA at 1:200, but not with paraffin oil. Exposing larvae to BA for 5 min alone did not affect sugar preference. Control larvae received no RFRP training. In all panels, numbers above the boxes indicate sample sizes. *n.s.* not significant, ^*^P < 0.05, ^**^P < 0.01, ^***^P < 0.001, Kruskal-Wallis test and *post hoc* Dunn’s multiple comparison for **A**, **C**, **E**, and **F**; Mann-Whitney test for **B**, ANOVA and *post hoc* Tukey’s multiple comparison test for **D**, and Fisher’s Exact test for **G**.

We assessed the behavioral effects of reward deprivation in *Drosophila* larvae by repeatedly pulling a 3rd instar larva away when it was about to reach a drop of yeast solution. Interestingly, we found that after about four approach-and-pull-away trials, wild-type larvae usually gave up further attempts to reach the yeast (Fig. 1A). If second or third rounds of approach-and-pull-away tests were repeated after 10 min, the larvae gave up more quickly, usually within two trials. This suggests that larvae become less resistant to failure (Fig. 1A). Interestingly, larvae that had given up pursuing the yeast also showed reduced light avoidance compared with larvae that had not received training (Fig. 1B). Thus we reasoned that repeatedly preventing the larva from accessing yeast prompted it to abandon the pursuit and demonstrate altered behavioral phenotypes. For convenience, we named the procedure larval RFRP (repeated failure in reward pursuit) training and the larvae that gave up further pursuit as RFRP larvae. The related behavioral phenotypes in RFRP larvae were then called RFRP phenotypes.

We next tried using BA to replace yeast as the source of attractive olfactory stimuli for RFRP training, as yeast components are complex (Fig. S1A, B). Instead of pulling the larva away from the attractant, we pushed the BA-container away from the larva when it was about to reach it, to avoid unnecessary disturbance to the larva (Movie S1; also see Methods for details). We tried BA dissolved in paraffin oil at ratios of 1:50, 1:100, 1:200, 1:300, and 1:400. We chose a BA dilution of 1:200 for standard RFRP training because at this concentration larvae made the most attempts to reach the BA before giving up (Fig. 1C).

We investigated four aspects of the larval RFRP-related phenotypes. Trial number before giving up was used to measure the development of RFRP states. The larger the trial number, the more resistant the larvae were to developing RFRP states. The other three aspects, decrease in velocity, decrease in light avoidance, and decrease in sugar preference, were used to measure the behavioral consequences of RFRP. Using 1:200 BA for RFRP training, wild-type larvae usually gave up odor-chasing after about four trials, similar to that seen during yeast training (Fig. 1A, C). Conversely, larvae pursued the stimulus for no or one trial only when the solvent paraffin oil alone was used for RFRP training. RFRP model larvae trained with BA also showed a decrease in velocity (Fig. 1D). As RFRP might have comprehensive behavioral consequences such as reduced preference for a sweet taste and less food and water intake, we also tested whether the innate preference behaviors in RFRP larvae were affected. We first tested them using a light-avoidance assay. A decrease in light avoidance was also seen in BA-trained RFRP larvae (Fig. 1E). Surprisingly, this decrease lasted for >60 min (Fig. 1F). Furthermore, RFRP larvae showed greatly reduced preference for 0.5 mol/L sucrose (Fig. 1G). Notably, larvae without training or subjected to training with paraffin oil did not show any change in velocity, light avoidance, or sugar preference (Figs. S1C–E and 1G), suggesting that the reduction in velocity, light avoidance, and sugar preference was not caused by our experimental manipulations. We also attempted to exclude the possible effect of BA exposure on larval velocity, light avoidance, and sugar preference. As larval RFRP training generally took ~5 min, we exposed the larvae to BA for 5 min and found no significant change in larval velocity, light avoidance, or sugar preference (Fig. 1D, E, G). For simplicity, we measured only the change in velocity and light avoidance but not sugar preference in RFRP larvae in the subsequent experiments.

### Octopamine is Required for Larval RFRP Phenotypes

**Fig. 2:**
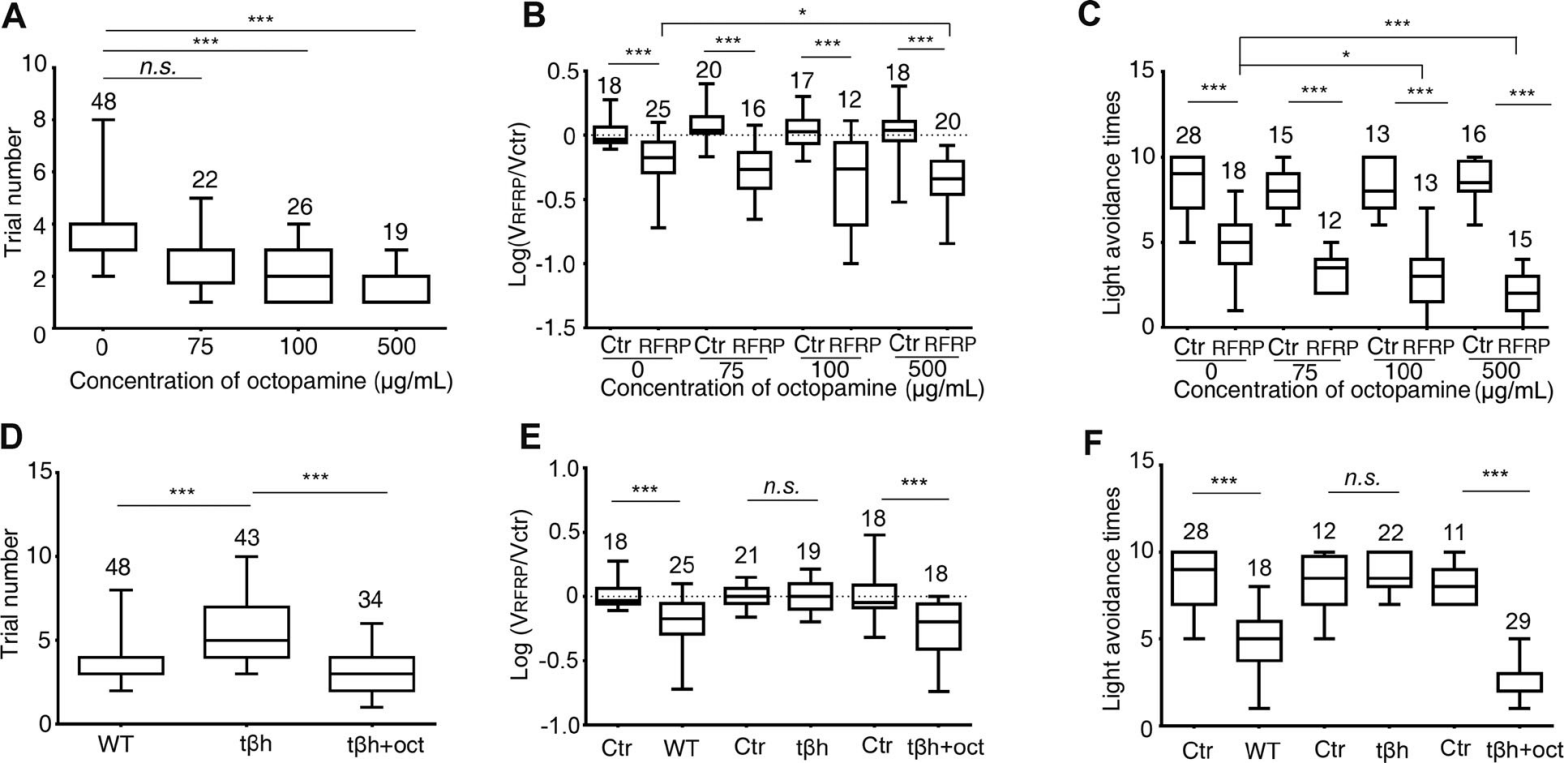
Octopamine positively regulates larval RFRP phenotypes. **A–C** Effects of different concentrations of octopamine on (**A**) trial number in RFRP training, (**B**) velocity, and (**C**) light avoidance after RFRP training. **D–F** 100 μg/mL octopamine restored (**D**) the increased trial number in RFRP training, (**E**) the abolished decrease in velocity, and (**F**) the abolished decrease in light avoidance in tβh mutants to the level of wild-type controls. For all panels, numbers above the boxes indicate sample sizes. ctr, control without RFRP training; *n.s.* not significant; ^*^P <0.05, ^**^P < 0.01, ^***^P < 0.001, Kruskal-Wallis test and *post hoc* Dunn’s multiple comparison for **A** and **D**; *t*-test for **B** and **E**; Mann-Whitney test for **C** and **F**.

To identify the molecules involved in the larval RFRP phenotypes, we performed LC-MS in larval brain before and after RFRP training to determine which molecules had changed. Four biogenic amines, dopamine, serotonin, tyramine and octopamine, were tested. Only octopamine demonstrated an up to 700% increase in concentration while changes in the remaining molecules were all less than 100% in RFRP larvae as compared with larvae exposed to BA (Fig. S2). Since norepinephrine is known to mediate mood modulation in vertebrates, we focused on the possible involvement of octopamine in the larval RFRP phenotypes. Octopamine at 100 and 500 μg/mL significantly reduced the trial number in RFRP training (Fig. 2A), suggesting that it facilitates the development of RFRP states. The trial number decreased as the octopamine concentration increased. Octopamine enhanced the decrease in larval velocity at the relatively high concentration of 500 μg/mL but not at lower concentrations (Fig. 2B). The decrease in light avoidance was significantly enhanced by octopamine at 100 and 500 μg/mL, but not at 75 μg/mL (Fig. 2C). These results suggested that octopamine-feeding facilitates the development of RFRP states and enhances the RFRP phenotypes at high concentrations.

We then used the tβh mutant, which is defective in the synthesis of octopamine from tyramine [26, 27], to determine whether loss of octopamine has an RFRP-antagonizing effect. Compared with wild-type controls, tβh mutants were less likely to develop RFRP phenotypes as the trial number in RFRP training increased (Fig. 2D). The trial number in the tβh mutant larvae was restored to the level of wild-type controls by feeding the larvae 100 μg/mL octopamine (Fig. 2D). The decrease in both velocity and light avoidance after RFRP was also abolished in the tβh mutants (Fig. 2E–F), but was effectively restored by supplementation with 100 μg/mL octopamine (Fig. 2E–F). It should be noted that although locomotor speed was impaired in the tβh mutant [27–29], the light-avoidance performance appeared to be unaffected (Fig. 2F). Together, these results show that octopamine is necessary for the development of RFRP states and the display of RFRP phenotypes. Excessive octopamine facilitated the development and enhancement of RFRP phenotypes.

### A Small Group of Octopaminergic Neurons is Required for Larval RFRP Phenotypes

**Fig. 3:**
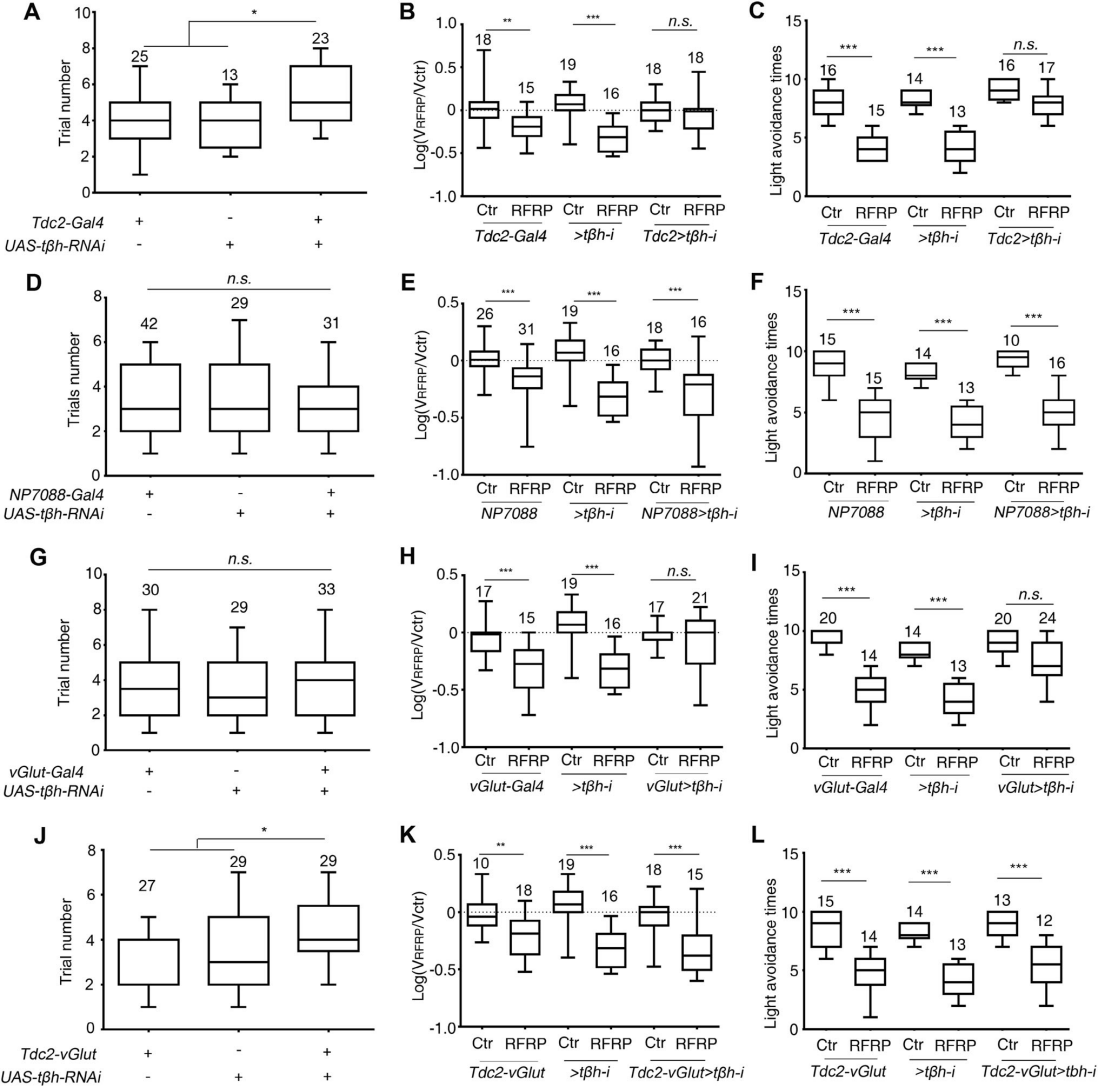
Downregulating tβh in different sets of CNS neurons affects distinct aspects of larval RFRP phenotypes. **A–L** Effects of knocking down tβh expression on trial number in RFRP training, the decrease in velocity, and the decrease in light avoidance after RFRP training with (**A**–**C**) *Tdc2-Gal4*, (**D**–**F**) *NP7088-Gal4*, (**G**–**I**) *vGlut-Gal4*, and (**J**–**L**) *Tdc2-Gal4* in combination with *vGlut-Gal80.* In all panels, numbers above the boxes indicate sample sizes. ctr, control without RFRP training. *>tβh-I* indicates *UAS-tβh–RNAi* and *Tdc2-vGlut* indicates *Tdc2-Gal4;vGlut-Gal80*. *n.s.*, not significant; ^*^P < 0.05; ^**^P < 0.01, ^***^P < 0.001, Kruskal-Wallis test and *post hoc* Dunn’s multiple comparison for **A**, **D**, **G**, and **J**; *t*-test for **B**, **E**, **H**, and **K**; Mann-Whitney test for **C**, **F**, **I**, and **L**.

As octopamine was a key role player in the larval RFRP-related phenotypes, we next considered which octopaminergic neurons might be involved in this process. Consistent with the tβh mutant phenotypes, knocking down tβh in octopamine-synthesizing neurons labeled with *Tdc2-Gal4* [18, 28, 30] led to increased trial numbers in RFRP training compared with the parental control lines (Fig. 3A) and abolishment of the decreases in velocity and larval light avoidance (Fig. 3B, C). We next tried to narrow down the scope of the candidate neurons. We knocked down *tβh* expression with *NP7088-Gal4*, which shares expression patterns in common with *Tdc2-Gal4* [30]. However, no change in trial number in RFRP training or larval RFRP phenotypes was seen (Fig. 3D–F). Intriguingly, driving the expression of *tβh-RNAi* with *vGlut-Gal4* completely abolished the decrease in velocity and light avoidance (Fig. 3H–I) but not that in trial number (Fig. 3G). In contrast, knocking down *tβh* expression with *Tdc2-Gal4* in combination with *vGlut-Gal80* improved the trial number (Fig. 3J) but had no effect on the decreases in velocity and light avoidance (Fig. 3K, L). Thus, the development of RFRP states and the behavioral exhibition of RFRP phenotypes might be separately regulated. Octopaminergic neurons that are also glutamatergic mediate the behavioral display of larval RFRP phenotypes, whereas non-glutamatergic neurons regulate the development of larval RFRP states.

**Fig. 4:**
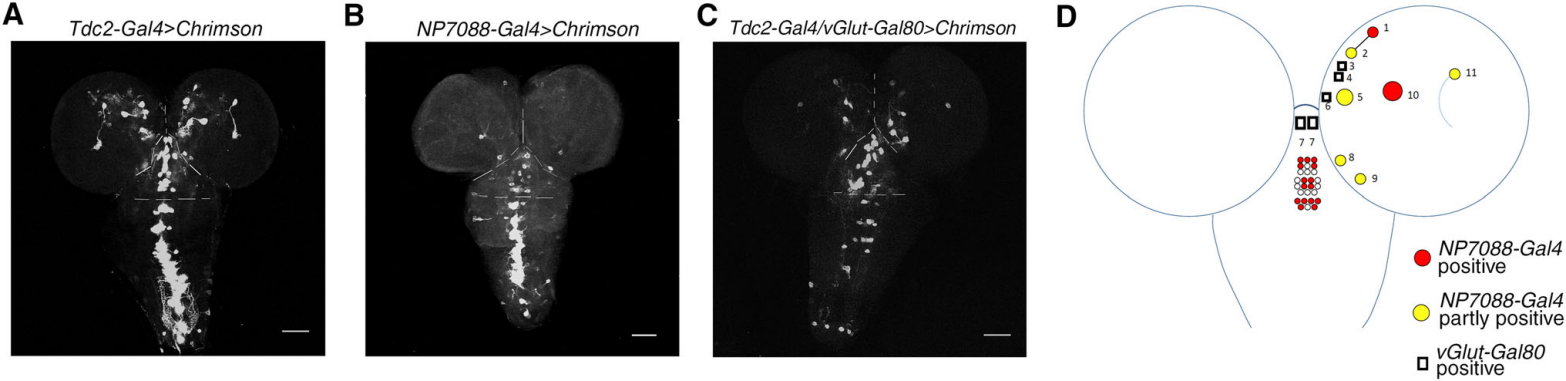
Neurons related to different aspects of larval RFRP phenotypes map to different subgroups in the central brain. **A–C** Expression patterns of *Tdc2-Gal4* (**A**), *NP7088-Gal4* (**B**), and *Tdc2-Gal4*; *vGlut-Gal80* (**C**) as indicated by *UAS-Chrimson*. Stack layers containing cell body signals were selected for better visualization of neurons. Scale bars, 50 μm for **A**–**C**. Dashed lines demarcate hemispheres, SOG, thoracic VNC, and abdominal VNC. **D** Schematic representation of larval RFRP-related neurons in a hemisphere and the SOG. Circles/rectangles, cell bodies of neurons labeled by *Tdc2-Gal4* in brain and SOG (only one hemisphere is indicated). Red, neurons positive for *NP7088-Gal4*; yellow, neurons partly positive for *NP7088-Gal4*. In the SOG segments, only the number but not the exact identity of neurons labeled by *NP7088-Gal4* is shown. Rectangles, neurons masked by *vGlut-Gal80*.

To narrow the scope of larval RFRP-related neurons, we compared the expression patterns of the above driver lines using *UAS-Chrimson*. Aside from neurons in the ventral nerve cord (VNC), *Tdc2-Gal4* labeled ~11 neurons in each brain hemisphere (Fig. 4A, D and Table 2). It also labeled one pair of neurons in the anterior-most part of the SOG that had not been previously described [29], 9 neurons (8.4 ± 0.22, *n* = 10 flies) in the mandibular segment, 10 neurons (9.2 ± 0.33, *n* = 10) in the maxillary segment, and 7 neurons (7.0 ± 0.0, *n* = 10) in the labial segment of the SOG (Fig. 4A, D and Table 3). These findings were similar to but slightly different from previous reports, probably because the *Tdc2-Gal4* lines used were different [29]. *NP7088-Gal4* seemed to label almost all of the *Tdc2-Gal4*-positive neurons in the thoracic and abdominal segments of the VNC (Fig. 4A, B). Thus, larval RFRP seems not to involve *Tdc2-Gal4*-labeled neurons in the VNC. This conclusion was supported by the results from *0665-Gal4*. This Gal4 labels most if not all of the octopaminergic neurons in the VNC but only a few in the hemispheres and SOG (Fig. S3A, Tables 2, 3). Knocking down *tβh* expression using this Gal4 did not change the trial numbers in RFRP training or the decreases in velocity and light avoidance after RFRP training (Fig. S3B–D). With regard to *Tdc2-Gal4* neurons in the hemispheres and SOG, *NP7088-Gal4* consistently labeled one medial neuron and one calyx neuron in each hemisphere, almost all neurons (6.1 ± 0.31, *n* = 10) in the labial segment, and some of the neurons in the mandibular (5.1 ± 0.41, *n* = 10) and maxillary (3.7 ± 0.42, *n* = 10) segments that were difficult to identify precisely (Tables 2, 3 and Fig. 4D). Thus, *Tdc2-Gal4* neurons in the hemispheres and in the mandibular and maxillary segments of the SOG, but not those in the VNC and the labial segment, were candidate regulators of the larval RFRP phenotypes. We next used *UAS-Chrimson* to visualize the repressive effect of *vGlut-Gal80* on *Tdc2-Gal4* in the hemispheres and SOG. *vGlut-Gal80* seemed to mask *Tdc2-Gal4* expression in about three medial neurons in each hemisphere and the one anterior-most pair of neurons in the SOG (Fig. 4A, C, D and Tables 2, 3). Thus, the four pairs of brain and SOG neurons masked by *vGlut-Gal80* were probably associated with the larval RFRP phenotypes. The rest of the mandibular and maxillary neurons, plus three pairs of neurons in the hemispheres, that were negative in *NP7088-Gal4* and not masked by *vGlut-Gal80* were likely associated with the development of larval RFRP states (Fig. 4D).

**Table 2:** Statistics of neuron labeling in driver lines in each larval hemisphere.

Neuron ID	*Tdc2-Gal4* (*n* = 10)	*NP7088-Gal4* (*n* = 10)	*Tdc2-Gal4/vGlut-Gal80* (*n* = 10)	*0665-Gal4* (*n* = 10)
1	1.0 ± 0.0	1.0 ± 0.0	1.0 ± 0.0	1.0 ± 0.0
2	1.0 ± 0.0	0.4 ± 0.16	0.8 ± 0.13	0.0 ± 0.0
3	1.0 ± 0.0	0.0 ± 0.0	0.1 ± 0.10	0.0 ± 0.0
4	1.0 ± 0.0	0.0 ± 0.0	0.2 ± 0.13	0.0 ± 0.0
5	1.0 ± 0.0	0.5 ± 0.17	0.8 ± 0.13	0.2 ± 0.20
6	0.9 ± 0.10	0.0 ± 0.0	0.1 ± 0.10	0.0 ± 0.0
7	1.0 ± 0.0	0.1 ± 0.33	0.2 ± 0.13	0.0 ± 0.0
8	1.0 ± 0.0	0.6 ± 0.16	1.0 ± 0.0	1.0 ± 0.0
9	0.9 ± 0.10	0.6 ± 0.16	0.7 ± 0.15	0.6 ± 0.24
10	0.8 ± 0.13	0.9 ± 0.10	1.0 ± 0.0	0.6 ± 0.24
11	0.9 ± 0.10	0.4 ± 0.16	1.0 ± 0.0	0.0 ± 0.0

Numbers of neurons labeled are presented as mean ± SEM. Neuronal IDs as in Fig. 4D. A neuron with an average labeling number < 0.3 was considered not labeled by the driver.

**Table 3:** Statistics of driver labeling in larval SOG segments.

SOG segment	*Tdc2-Gal4* (*n* = 10)	*NP7088-Gal4* (*n* = 10)	*Tdc2-Gal4/vGlut-Gal80* (*n* = 10)	*0665-Gal4* (*n* = 5)
Mandibular	8.4 ± 0.22	5.1 ± 0.41	8.0 ± 0.39	1.6 ± 0.40
Maxillary	9.2 ± 0.33	3.7 ± 0.42	9.8 ± 0.13	3.4 ± 0.40
Labial	7.0 ± 0.0	6.1 ± 0.31	7.0 ± 0.0	5.0 ± 0.45

Numbers of neurons labeled are presented as mean ± SEM.

**Fig. 5:**
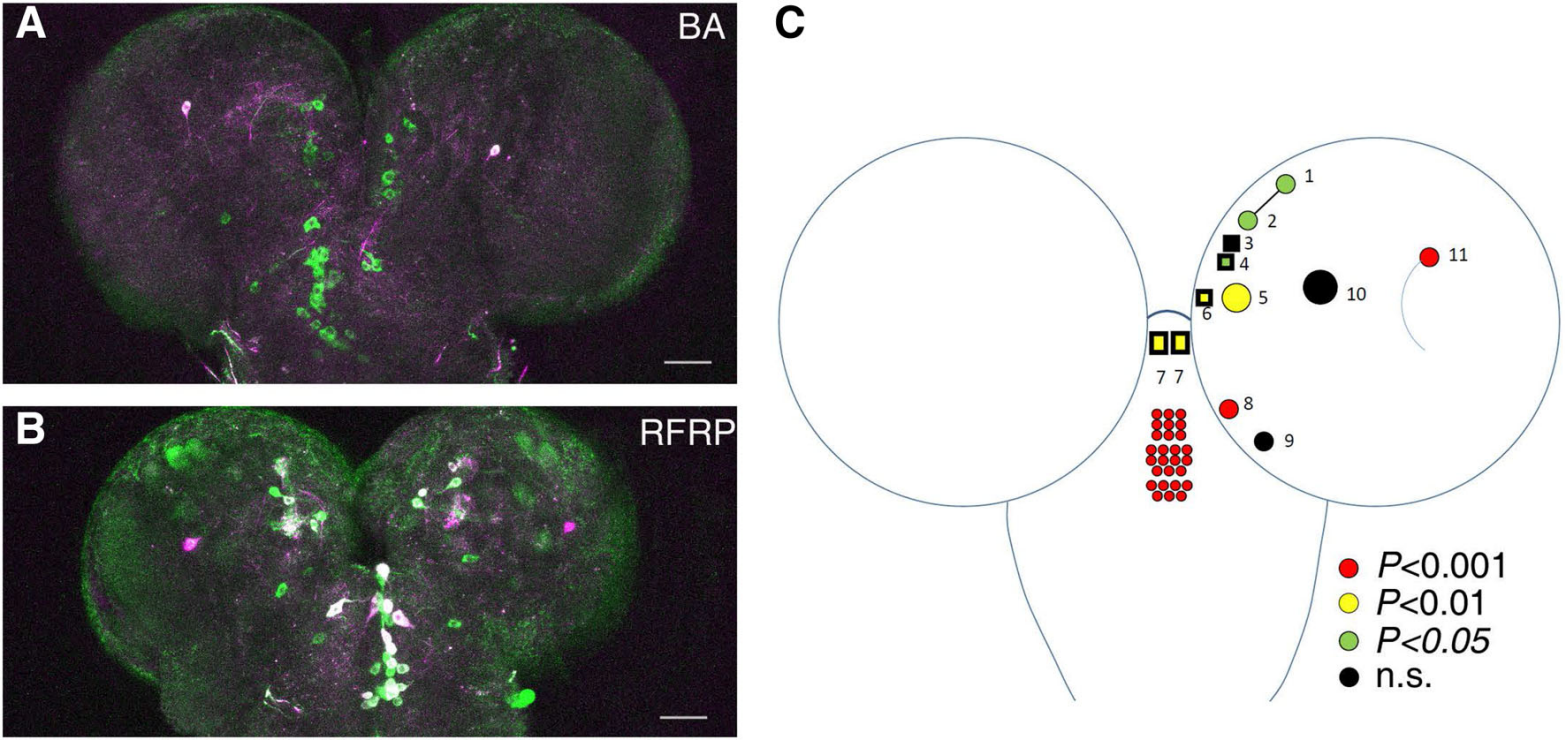
Photoconversion of CaMPARI in octopaminergic neurons by RFRP training. **A–B** *Tdc2-Gal4* was used to drive *UAS-CaMPARI* expression. Green and red signal respectively indicate CaMPARI before (**A**) and after (**B**) photoconversion. BA, samples subjected to 5 min BA exposure; RFRP, samples subjected to RFRP training. **C** Cartoon summarizing the statistics of CaMPARI photoconversion in Table 4.

The role of octopaminergic neurons in larval RFRP phenotypes can be further confirmed by imaging neuronal activity using a recently-developed Ca^2+^-dependent imaging tool, CaMPARI [24]. In control larvae exposed to BA for 5 min, activation of neurons as indicated by the photoconversion of CaMPARI from green to red was barely detectable in octopaminergic neurons associated with the candidate larval RFRP phenotypes (Fig. 5A, Table 4). In larvae that had received RFRP training, almost all the candidate RFRP-related octopaminergic neurons were activated after training except for three in each hemisphere (Fig. 5B, C, Table 4) as compared with larvae exposed to BA alone, supporting a role of octopaminergic neurons in larval RFRP phenotypes. Considering that all of the *Tdc2-Gal4* neurons in the SOG were positive for *tβh* immunoactivity and all those in the hemispheres were negative for *tβh* immunoactivity [29], these three pairs of hemisphere neurons can be largely excluded as regulators of larval RFRP phenotypes.

**Table 4:** Statistics of CaMPARI photoconversion measured by Log(F_red_/F_green_) in candidate larval RFRP-related octopaminergic neurons after BA exposure or RFRP training.

Name of neuron or SOG segment	BA exposure	RFRP training	*P* value	Significance of difference
1	−0.12 ± 0.05 *n* = 9	0.07 ± 0.05 *n* = 11	0.02	*
2	−0.24 ± 0.07 *n* = 8	0.02 ± 0.07 *n* = 9	0.02	*
3	−0.14 ± 0.04 *n* =12	−0.02 ± 0.07 *n* = 8	0.14	*n.s*
4	−0.10 ± 0.03 *n* = 9	0.04 ± 0.04 *n* = 6	0.02	*
5	−0.377 ± 0.07 *n* = 16	0.01 ± 0.05 *n* = 8	0.0018	**
6	−0.17 ± 0.03 *n* = 8	−0.01 ± 0.03 *n* = 11	0.0023	**
7	−0.22 ± 0.07 *n* = 6	0.09 ± 0.05 *n* = 8	0.0041	**
8	−0.20 ± 0.05 *n* = 7	0.002 ± 0.03 *n* = 11	0.0009	***
9	−0.21 ± 0.02 *n* = 5	−0.11 ± 0.11 *n* = 5	0.43	*n.s*
10	−0.50 ± 0.05 *n* = 12	−0.46 ± 0.06 *n* = 13	0.56	*n.s*
11	0.24 ± 0.04 *n* = 12	0.59 ± 0.03 *n* = 19	< 0.0001	****
Mandibular	−0.27 ± 0.04 *n* = 50	0.26 ± 0.21 *n* = 51	< 0.0001	****
Maxillary	−0.17 ± 0.19 *n* = 58	0.21 ± 0.23 *n* = 65	< 0.0001	****
Labial	−0.20 ± 0.12 *n* = 41	0.19 ± 0.21 *n* = 51	< 0.0001	****

Log(F_red_/F_green_), ratio of photoconversion of CaMPARI. Candidate larval RFRP-related octopaminergic neurons are numbered from 1 to 11; octopaminergic neurons in the SOG are quantified in groups as segments. Hemisphere neurons and SOG segments with no significant differences in Log(F_red_/F_green_) between BA and RFRP groups are indicated in red. ^*^P < 0.05, ^**^P < 0.01, ^***^P < 0.001, *t*-test. Data are presented as mean ± SEM. The results are also summarized as a cartoon in Fig. 5.

## Discussion

When repeatedly prevented from obtaining a reward, a *Drosophila* larva finally stops pursuing the attractant. Meanwhile, behavioral phenotypes such as decreased locomotor velocity and reduced innate preferences occur. If the deprivation of reward is considered as a form of punishment, our training process is similar to that of some learned-helplessness models, in which animals are subjected to repeated unavoidable punishment before they finally stop trying to escape [31–34]. In terms of the behavioral consequences, the reduced larval locomotor activity after training is similar to what is seen in other learned-helplessness models [31–34]. Our experiments provide a new model similar to learned helplessness. In addition, larval light avoidance and sugar preference, which involve the different sensory modalities of vision and gustation, were both undermined in RFRP larvae. This meant that repeated failure to receive a reward was able to repress larval performance in behavioral tasks that involve other sensory modalities, suggesting that RFRP training changes the internal state of the larvae. The RFRP model presents a new way to test how *Drosophila* larvae respond to failure in obtaining an expected reward.

Comparatively, octopamine showed the most significant change after RFRP training among all the candidate molecules tested. Similar to its vertebrate homologue norepinephrine, which is thought to be involved in emotion in vertebrates, octopamine released from a few neurons located in the larval brain was able to facilitate the RFRP-related phenotypes, probably in distinct manners. As octopamine is known to mediate appetitive sensory cues in learning and memory [18], it may serve as the molecule that signals reward in our RFRP model. When the octopamine level was decreased, for example in the *tβh* mutant, the same appetitive stimulation was less rewarding. Failure to receive the reward thus means less loss and thus less punishment. Consequently the larvae are more resilient to RFRP training. In addition, the reduced locomotor activity after RFRP training was not a direct result of elevated octopamine levels since octopamine stimulates larval locomotor activity [27, 28]. Our mapping of larval RFRP-related octopaminergic neurons also excluded those motor neurons in the VNC from being involved. Thus, the reduced locomotor activity must result from the internal state of the RFRP larva.

The candidate larval RFRP-regulated neurons were largely localized to the medial brain and anterior SOG region in our study. According to Selcho *et al.* [29], *Tdc2-Gal4* neurons in the hemispheres were all negative, while those in the SOG region were all positive for tβh and octopamine immunoactivity. As the larval RFRP phenotypes were clearly mediated by octopamine, the candidate neurons in the SOG are more likely the key regulators of larval RFRP phenotypes than those in the hemispheres. However, it is possible that the tβh and octopamine levels were low in the *Tdc2-Gal4* neurons in the hemispheres, making them difficult to detect using antibodies. Thus, the involvement of neurons in the hemispheres cannot be completely excluded. Notably, the functions of glutamatergic and non-glutamatergic Tdc2 neurons seemed to be different and non-overlapping: the former regulate RFRP behavioral phenotypes and the latter regulate the development of RFRP states. These two aspects are behaviorally related but mechanistically different.

Taken together, the larval *Drosophila* RFRP model provides a simplified framework for investigating the neural basis of how larvae deal with failure in obtaining an expected reward.

## Electronic supplementary material

Below is the link to the electronic supplementary material.

Supplementary material 1 (PDF 597 kb)

Supplementary material 2 (MP4 12333 kb)
