# Supplementary material for: Repeated Failure in Reward Pursuit Alters Innate Drosophila Larval Behaviors
Source: Neurosci Bull. 2018 Jun 27;34(6):901–11. doi: 10.1007/s12264-018-0248-0 (PMC6246844; doi:10.1007/s12264-018-0248-0)
Supplement: Supplementary file 1 — Supplementary material 1 (PDF 597 kb) [file 12264_2018_248_MOESM1_ESM.pdf]

Supplemental Materials:

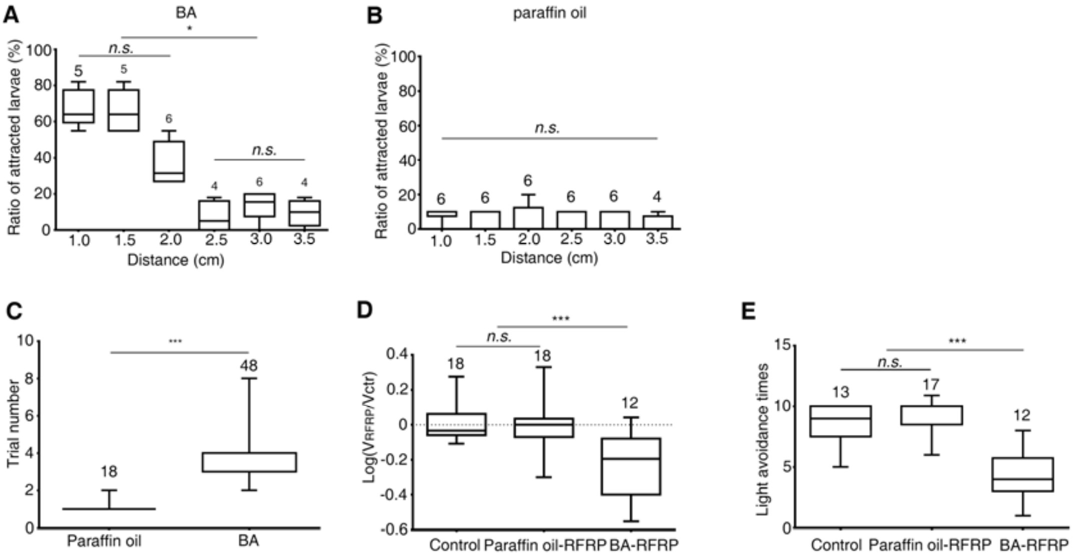

Fig. S1 Larva RFRP phenotypes can be induced by training with BA. Related to Fig. 1.

A–B BA was Attractive to Larva within 2.5 cm. **A** Ratio of larvae attracted by BA dissolved in paraffin oil at 1:200 at various distances from the BA container. **B** Ratio of larvae attracted by paraffin oil at various distances. For all panels, numbers above the box indicate sample sizes. *n.s.* not significant, $^{*}P < 0.05$, *Kruskal-Wallis* test and post hoc *Dunn's* multiple comparison. **C–E** Larval RFRP phenotypes can be induced by training with BA but not paraffin. **C** Trial number of larvae in RFRP training with paraffin oil or paraffin oil containing 1:200 BA. **D** Decrease in larval velocity after RFRP training with paraffin oil or paraffin oil containing 1:200 BA. **E** Decrease in larval light avoidance after RFRP training with paraffin oil or paraffin oil containing 1:200 BA. For all panels, numbers above the box indicate sample sizes. Control groups received no RFRP training; *n.s.* not significant, $^{***}P < 0.001$, *Kruskal-Wallis* test and post hoc *Dunn's* multiple comparison for **A**, **B**, **E**; Mann-Whitney test for **C**; *one-way* ANOVA and post hoc *Tukey's* multiple comparision test for **D**.

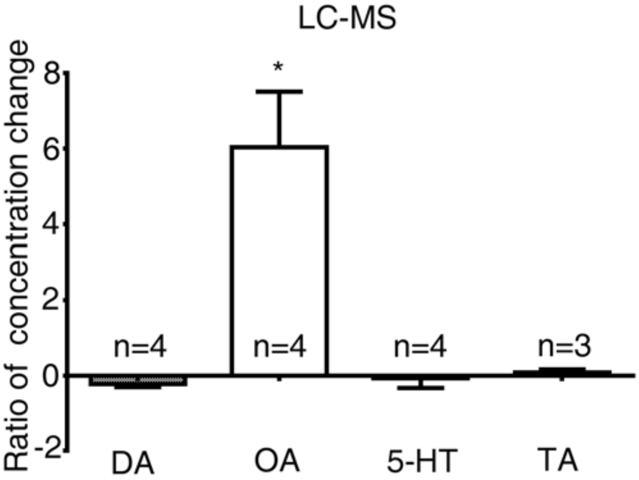

Fig. S2 Ratio of concentration after larval RFRP training to that before RFRP training as measured using LC-MS. Related to Fig. 2. OA concentration increase is significantly above zero. Data are presented as mean±s.e.m. *, *P*<0.05, *t*-test.

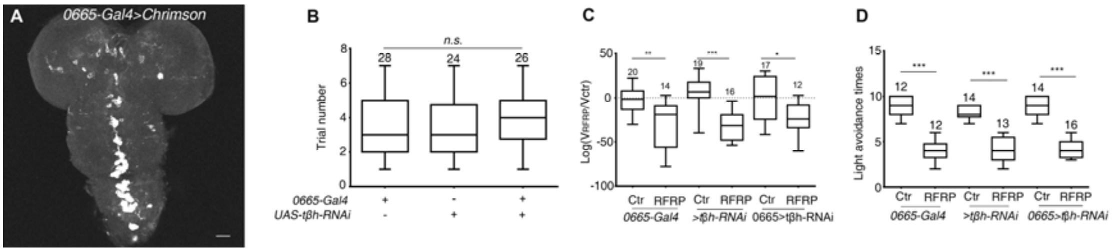

Fig. S3 *0665-Gal4* labelled neurons are dispensable for larval RFRP phenotypes. Related to Fig. 3 and Fig. 4. **A** Expression pattern of *0665-Gal4* in larval CNS. **B–D** Knocking down tβh expression with *0665-Gal4* does not affect trial number in larval RFRP training **(B)**, and decrease in locomotion speed **(C)** and light avoidance **(D)** after RFRP training. Scale bar is 20 μm in **A**. For **B–D**, numbers above the box indicate sample sizes. ctr indicates control that received no RFRP training. *>tβh-i* indicates *UAS-tβh–RNAi.* n.s. not significant, $^{*}P < 0.05$, $^{**}P < 0.01$, $^{***}P < 0.001$, *Kruskal-Wallis* test for **B**; *t*-test for **C**; *Mann-Whitney* test for **D**.

Movie S1 Video of a wild type larva in RFRP training with BA. BA is in the white container. Related to Fig. 1.
